# The fetal programming effect of maternal immune activation (MIA) on the offspring’s immune system

**DOI:** 10.1007/s00281-024-01023-8

**Published:** 2024-08-30

**Authors:** Naomi Hofsink, Lucianne Groenink, Torsten Plösch

**Affiliations:** 1 Department of Obstetrics and Gynaecology, University Medical Center Groningen, University of Groningen, Groningen, The Netherlands; 2 Department of Pharmacology, Utrecht Institute for Pharmaceutical Sciences, Utrecht University, Utrecht, The Netherlands; 3 Carl von Ossietzky Universität Oldenburg School VI - School of Medicine and Health Sciences, Department of Paediatrics, Section of Neonatology, and Research Centre Neurosensory Science, Oldenburg, Germany

**Keywords:** Fetal programming, Maternal immune activation, Immune development, Immune function

## Abstract

The first 1000 days of life is a critical period of development in which adverse circumstances can have long-term consequences for the child’s health. Maternal immune activation is associated with increased risk of neurodevelopmental disorders in the child. Aberrant immune responses have been reported in individuals with neurodevelopmental disorders. Moreover, lasting effects of maternal immune activation on the offspring’s immune system have been reported. Taken together, this indicates that the effect of maternal immune activation is not limited to the central nervous system. Here, we explore the impact of maternal immune activation on the immune system of the offspring. We first describe the development of the immune system and provide an overview of reported alterations in the cytokine profiles, immune cell profiles, immune cell function, and immune induction in pre-clinical models. Additionally, we highlight recent research on the impact of maternal COVID-19 exposure on the neonatal immune system and the potential health consequences for the child. Our review shows that maternal immune activation alters the offspring’s immune system under certain conditions, but the reported effects are conflicting and inconsistent. In general, epigenetic modifications are considered the mechanism for fetal programming. The available data was insufficient to identify specific pathways that may contribute to immune programming. As a consequence of the COVID-19 pandemic, more research now focuses on the possible health effects of maternal immune activation on the offspring. Future research addressing the offspring’s immune response to maternal immune activation can elucidate specific pathways that contribute to fetal immune programming and the long-term health effects for the offspring.

## Introduction

The first 1000 days of life, from conception to two years after birth, are an important period in human life characterized by rapid development of organs and tissues. This period lays the foundation for the child’s overall health and developmental trajectory. However, it can be influenced by the environment [1, 2]. Adverse circumstances, like infection, during this critical period can have long-term consequences for the child’s health. Permanent deviations in the structure, physiology, and metabolism of organs and tissues may take place depending on severity and timing, which could result in long-term changes in organ function [1–3]. These permanent adaptations can predispose the child to developing cardiovascular, metabolic, allergic, and autoimmune diseases. This process is referred to as “fetal programming” or the “Developmental Origins of Health and Disease” paradigm (DOHaD) [1–3]. Alterations to epigenetic markers, which are crucial in determining the transcription of genes, are a method by which prenatal exposures can predispose to later long-term health consequences [4, 5].

During pregnancy, the precise and balanced regulation of the immune system plays an important role in a healthy pregnancy. To support and continue a healthy pregnancy, immune cells and cytokine signaling pathways take part in the coordinated communication between mother and child. Depending on the stage of pregnancy, dynamic alterations occur in the maternal and fetal immune responses [6, 7]. The mother’s immune system must protect against pathogens and be able to tolerate and prevent immunological-mediated harm to the fetus. Interferons (IFNs) play an important role in pregnancy and development. At the same time, IFNs also play a role in the immune response as a defense against pathogens [8]. Therefore, alterations in the immune response during pregnancy may lead to pregnancy complications and fetal defects.

Besides adjustments to the immune system of the mother, the immune system of the fetus is also developed. The fetal immune system of the fetus not only develops during the first 1000 days of life, but also reacts to environmental stimuli. The uterus is the first environment the fetal immune system encounters. Despite being physically separated from the mother by the placental membrane, various factors like hormones and cytokines provide environmental cues to the developing fetus [9]. Therefore, it is likely that the maternal immune system may have long term effects on the fetal immune system. Furthermore, the gene expression profile of all the immune cells is controlled in a cell- and lineage-specific manner. Epigenetic programming of the immune cells is important for the maintenance, tolerance, training, and memory of the immune response. Alterations in the epigenetic landscape may have lasting implications for long-term health [10, 11].

Activation of the maternal immune system due to an infection can affect the delicate immune balance during this critical period of development. For over 30 years, an increasing body of epidemiological evidence has accumulated suggesting an association between maternal infection, maternal immune activation (MIA), and the increased risk of neurodevelopmental disorders [12–27]. MIA is linked, independent on specific pathogens, to an increased risk of schizophrenia [13, 16, 18–20, 23] and autism spectrum disorder (ASD) [15, 21–24, 26]. Moreover, immune system and immune response abnormalities are frequently observed in neurodevelopmental and neuropsychiatric disorders [28–33]. There is a high incidence of aberrant immune responses reported in individuals with autism and schizophrenia [23, 28], including increased numbers of monocytes [29, 34] and alterations in the plasma cytokine profile [31, 32, 35].

The impact of MIA on the short- and long-term health of the offspring can be studied with animal models. In these models, a stimulus that mimics an infection triggers the maternal immune system, which causes cytokine production and immunological alterations that also impact the fetus [36]. Viral mimic polyinosinic: polycytidylic acid (poly(I: C)) and bacterial mimic lipopolysaccharide (LPS) are commonly used to induce MIA in models. The association between MIA and the risks of neurodevelopmental disorders, such as schizophrenia and ASD, is the main focus of these preclinical studies [37]. However, the effects of MIA are not limited to the central nervous system: effects of MIA on the offspring’s metabolism, immune system, circulation, and reproduction have also been reported [37]. Human epidemiologic research indicates that MIA results in several disorders associated with aberrant immunity in the offspring, including type 1 diabetes, hypersensitivity in allergic diseases, asthma, and immune overreaction in neurodevelopmental disorders [38–42].

The term MIA in its current meaning was first introduced in 2005 [43]. In general, it refers to an immune response during pregnancy resulting from maternal exposure to any inflammatory factor. The inflammatory factors inducing MIA include bacterial, viral, or fungal infection, stress, and metabolic disorders. Here we focus on the effect of MIA, induced by a viral or bacterial infection, on the immune system of the offspring.

## Development of the immune system

**Fig. 1:**
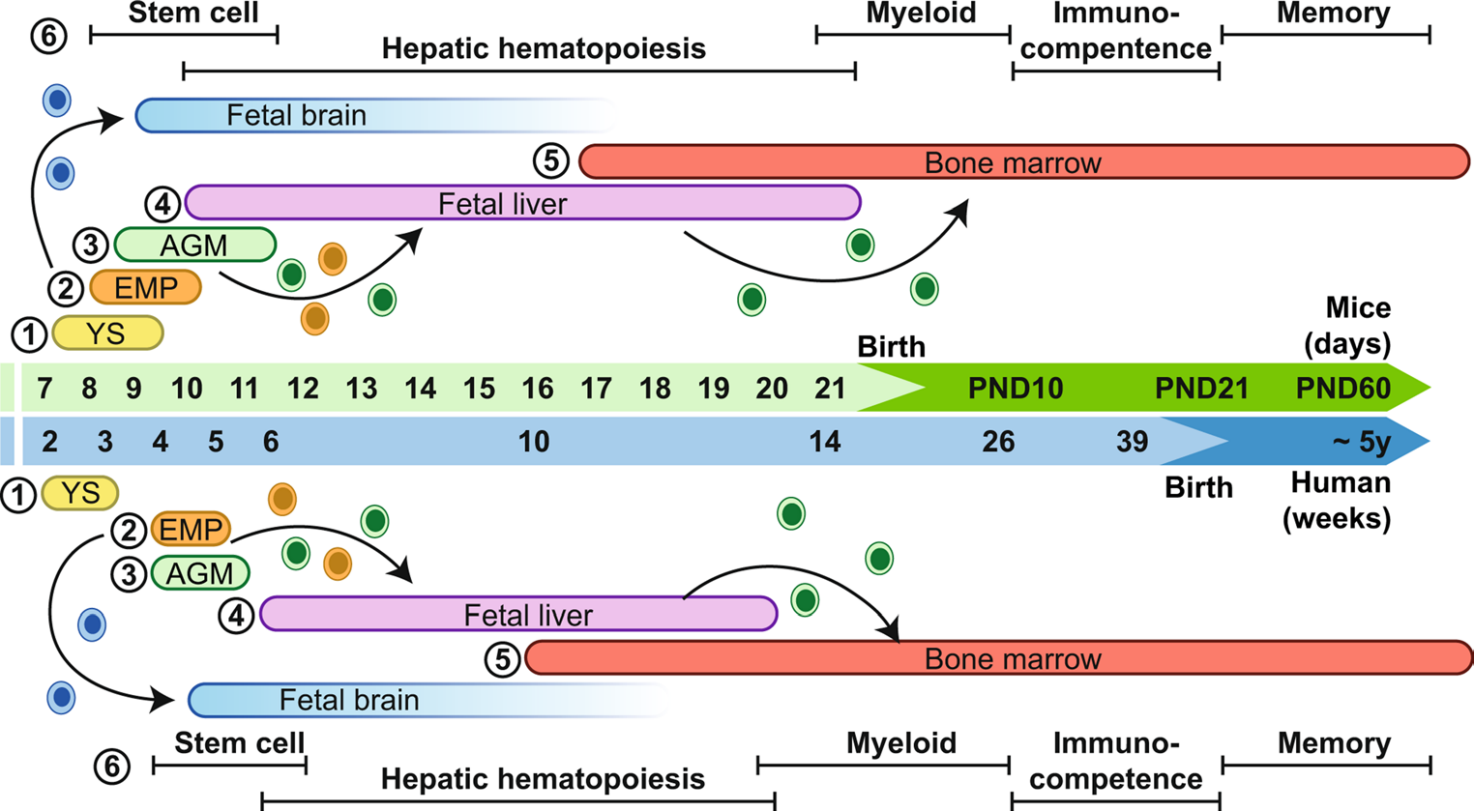
The developmental timeline of the hematopoietic system in mice and humans. The development of the hematopoietic system consists of three waves. **(1)** The primitive hematopoiesis wave, generated from the yolk sac (YS), gives rise to primitive erythroid progenitors to support the developing embryo. **(2)** The second wave gives rise to erythro-myeloid progenitors (EMP). The progenitors migrate to the fetal liver, the primary site for hematopoiesis, proliferate and differentiate in definitive erythrocytes, macrophages, and other myeloid lineages. The EMPs are also the progenitors of the microglia, primitive macrophages migrate to the brain before the blood-brain barrier is established around GD13. **(3)** The *de novo* definitive hematopoiesis gives rise to the definitive HSCs, originating from the AGM region. **(4)** The definitive HSCs migrate to the fetal liver to undergo proliferation and differentiation. **(5)** The HSCs and progenitors migrate to the bone marrow, where they are maintained throughout adulthood. **(6)** The critical windows for the immune development are the stem cell, hepatic hematopoiesis, myeloid, immunocompetence and memory periods. YS; yolk sac, EMP; erythro-myeloid progenitors, AGM; aorta-gonad-mesonephros, HSCs; hematopoietic stem cells, PND; postnatal day. Figure modified from Soares-da-Silva [46] and Veru [44]

The immune system develops in a similar manner in all mammals, with a sequential progression of events. Extrapolation between species is possible since the mature state of the immune system in mammals is similar for organs, cells, receptors, cytokines, intracellular messengers, and transcriptional factors [44]. During development, the receptor profile of the immune cells changes, which modifies how receptive they are to external stimuli, including cytokines and hormones. This results in distinct periods, the critical windows of development, during which different cellular components of the immune system are sensitive to these environmental influences (Fig. 1) [44–46].

### Critical windows of development for the immune system

The development of the hematopoietic system in mice and humans is reviewed by others [44–47]. Here we discuss, in short, the important stages and the five critical windows of development described by Veru and colleagues (Fig. 1) [44]. Fetal hematopoiesis is located at several sites during development to accompany the changes in the anatomy of the growing embryo and to help with the development of the embryo. In the first wave of hematopoiesis, known as primitive hematopoiesis, primitive erythroid progenitors are generated from the yolk sac and support the needs of the embryo in the first developmental stages [46, 48–50]. The second wave of hematopoiesis gives rise to yolk sac-derived lympho-myeloid and erythro-myeloid progenitors (EMPs), which migrate to the fetal liver. The fetal liver is the primary site of hematopoiesis during development [46, 47, 50, 51]. The EMPs are also the progenitors of the microglia, a distinct subset of macrophages residing in the central nervous system. Primitive macrophages migrate to various tissues, including the brain. The primitive macrophages, that migrated to the brain give rise to the future microglia cells. These primitive macrophages migrated to the brain before the blood-brain barrier (BBB) is established, which is around GD13.5 [52–54]. Simultaneously, the third wave of hematopoiesis produces *de novo* definitive hematopoietic stem cells (HSCs), which originate from the aorta-gonad-mesophros (AMG) region. The HSCs migrate to the fetal liver, where they undergo proliferation and differentiation. The downstream progenitors in the fetal liver are responsible for seeding the other hematopoietic organs [46, 47, 50, 51, 55–57].

In the first window of critical development, the HSCs are generated, which occurs in the second and third wave of hematopoiesis [44]. The second window of critical immune development begins with the start of hepatic hematopoiesis. In this period, the initial pool of T and B lymphocytes undergoes gene rearrangement and acquires antigen specificity [44]. The switch from hepatic to bone marrow hematopoiesis initiates the third window of critical immune development. In this period, from birth until postnatal day (PND)10 in mice and between 14 and 26 weeks in humans, there is an expansion of the lymphocytic cell populations. At the end of this window, the immune system in nearly fully developed [44]. In the fourth window of critical immune development, the immunocompetence window, the immune system undergoes functional maturation. The innate immune system gains functional abilities, followed by the functional ability of the adaptive immune system. In humans, the adaptive immune system gains functional ability at the end of the third trimester, while in mice, it occurs around PND21 [44]. In the last window of critical development, the immune system completes maturation. The functional naïve immune system will gain immunological memory in this period to create a faster and stronger response to microorganisms. The development of this memory takes time. In humans, this period begins at birth and lasts until approximately the age of five. This period in the mice lasts until adulthood (PND60) [44].

### Response of HSCs to cytokines

The HSCs respond to cytokines produced both locally and distantly, such as pro-inflammatory cytokines and chemokines. The HSCs can skew the differentiation towards the myeloid lineage in response to an infection, at the expense of lymphopoiesis and erythropoiesis [51, 58, 59]. Moreover, HSCs can produce cytokines to promote inflammation and assist myeloid and lymphoid cell development [58, 60]. Prolonged exposure to pro-inflammatory cytokines has an impact on HSCs. Chronic infection has been linked to a functional decline in HSCs, including a decreased repopulating ability and an increase in proliferation to the myeloid lineage. Additionally, pro-inflammatory cytokines have an indirect and direct effect on the HSCs, leading to secondary inflammatory signals produced by the bone marrow niche cells [60, 61].

In summary, adult HSCs are generated mid-gestation in mice and in the first trimester in humans, which is the first window of critical development. The HSCs undergo rapid proliferation in the second window of critical development before relocation to the bone marrow. The end of the second critical window of development is around birth in mice and at the end of the first trimester in humans. Exposure to adverse circumstances during these windows, like pro-inflammatory cytokines from maternal immune activation, might influence the function of the HSCs and multipotent progenitor, resulting in alterations in immune function in later life. Furthermore, during the last three windows of vulnerability, the functional development and maturation of the immune system take place. Adverse circumstances and environmental factors during these windows can also influence the immune response in later life, most probably by affecting the progenitor cells.

## Preclinical models of MIA: the programming effect on the immune system

Over the past years, multiple studies have analyzed the possible fetal programming effects of maternal infection and MIA on the offspring’s immune system. In MIA exposed offspring, alterations were found in cytokine profiles (Table 1 and online dataset), immune profiles, and immune cell function (Table 2). Most studies using preclinical MIA models concentrate on the association between MIA and neurodevelopmental disorders such as schizophrenia and autism spectrum disorder. Unsurprisingly, acute and long-term alterations of immune function have been studied in the offspring’s brain. Numerous studies have analyzed the cytokine profile in the brain of the offspring at various ages and brain regions (Table 1 and online dataset).

**Table 1:** Cytokine profile of MIA exposed offspring. Reported alterations in cytokine profile in different tissues or produced after in vitro stimulation from multiple studies. Cytokines reported were altered at specific age, with or without immune induction or at a specific part of the brain. A complete overview of the cytokine profile, including unaltered cytokines, can be found in the and online dataset. GD; gestational day, PND; postnatal day

Reference	Model	MIA induction		Age offspring	Tissue/ cell type	Cytokine	Notes
		GD	Method				
Meyer (2006) [79]	C57Bl/6J	GD9 & GD17	5 mg/kg poly(I: C)	GD9, GD17	Brain	Il-1β, Il-6, Il-10	3 h, 6 h after MIA. Behavioral alterations adult offspring.
Arrode-Brusés (2012) [80]	C57Bl/6J	GD16	20 mg/kg poly(I: C)	GD16	Brain	Il-1β, Il-7, Il-9, Il-10, Il-13, Il-13, Il-15, Il-17, Eotaxin, Mcp-1, Mig, Mip-1α, Mip-1β, GM-CSF, M-CSF, RANTES KC	6 h, 24 h after MIA.
Hsiao (2012) [77]	C57Bl/6J	GD12.5	20 mg/kg poly(I: C)	PND60	In vitro CD4 + T cells	Il-6, Il-17	Stimulated.
Krstic (2012) [66, 67]	C57Bl/6J	GD17	5 mg/kg poly(I: C)	PND20, PND30, PND450	Plasma	Il-1β	
			5 mg/kg poly(I: C)	PND450	Brain	Il-1α, Il-6, Il-9, Il-10	Hippocampus.
Garay (2013) [64]	C57Bl/6J	GD12.5	20 mg/kg poly(I: C)	PND0, PND7, PND14, PND30, PND60	Serum	Il-1α, Il-1β, Il-2, Il-3, Il-6, Il-12(p40), Il-12(p70), Ifnγ, Tnfα, KC, Mip-1α, Mip-1β, RANTES, GM-CSF, G-CSF	
					Brain	Il-1α, Il-1β, Il-2, Il-4, Il-5, Il-6, Il-9, Il-10, Il-12(p40), Il-12(p70), Il-13, Il-17, Ifnγ, KC, Mcp-1, Mip-1α, G-CSF, GM-CSF, Eotaxin, RANTES	Frontal-, Cingulate cortex, Hippocampus. Schizophrenia and ASD associated behavior.
Mandal (2013) [74]	C57Bl/6J	GD12.5	20 mg/kg poly(I: C)	PND60	Plasma	Il-6, Il-10	After acute inflammatory response.
					Peritoneal fluid	Il-6, Il-10, Tnfα	After acute inflammatory response.
Arsenault (2014) [63]	C57Bl/6J	GD15-17	5 mg/kg poly(I: C)	PND10	Serum	Il-2, Il-5, Il-6	
	C57Bl/6J	GD15-17	5 mg/kg poly(I: C) or 120ug/kg LPS	GD18	Brain	Tnfα, Mip-1β	Sensorimotor development delay.
Luan (2015) [73]	C57Bl/6J	GD12.5	50ug/kg LPS	PND60	Serum	Tnfα, Mip-1β	In vivo LPS-shock.
Pacheco-López (2013) [68]	C57Bl/6J	GD9	5 mg/kg poly(I: C)	PND30, PND70	Plasma	Il-2, Il-6, Tnfα, Ifnγ	
Onore (2014) [78]	C57Bl/6J	GD12.5	20 mg/kg poly(I: C)	PND70	In vitro BMDM	Il-1β, Il-12(p40), Mip-1α, Mip-1β	± stimulation LPS.
						Il-1β, Il-12(p40), Mip-1α	± polarization (M1/M2).
Giovanoli (2015) [69]	C57Bl/6J	GD17	5 mg/kg poly(I: C)	PND30, PND150, PND670	Plasma, Brain	No alterations in cytokine profile	Brain: Hippocampus
Giovanoli (2016) [67]	C57Bl/6J	GD9	5 mg/kg poly(I: C)	PND90	Plasma	Il-1β	
					Brain	Il-1β	Hippocampus. Prepulse inhibition deficits.
O’Loughlin (2017) [84]	C57Bl/6J	GD12.5	50ug/kg LPS	GD12, GD16, GD18, PND0, PND40	Brain	Il-1β, Il-6, Il-10, Tnfα, Mcp-1	PND7-PND40: Amygdala
Pendyala (2017) [82]	FVB/N Tg (Pcp2-EGFP) BT153Gsat/ Mmmh mice	GD12.5	20 mg/kg poly(I: C)	PND1, PND7, PND14, PND30	Brain	Il-2, Il-3, Il-6, Il-17, Tnfα	Cerebellum
Hsueh (2018) [65]	C57Bl/6J	GD15-17	25-50ug/kg LPS	PND35, PND56	Serum	Il-1β, Il-6, Il-10, Il-12(p40), Il-17a, Tnfα, Ifnγ, Mcp-1, Mig, Mip-1α, Mip-1β, RANTES	± Immune induction.
					Brain	Il-1β, Il-2, Il-4, Il-6, Il-10, Il-12(p40), Tnfα, Ifnγ, Mcp-1, RANTES	Social deficits adolescence and adulthood.
Carlezon (2019) [83]	C57Bl/6J	GD12.5	20 mg/kg poly(I: C)	PND90	Brain	Il-1β, Il-6, Il-10, Tnfα, Tgf-b1	Different brain regions. Anxiety-like behavior male offspring.
Wang (2019) [81]	C57Bl/6J	GD12.5	20 mg/kg poly(I: C)	GD14.5	Brain	Il-1β, Il-6	Anxiety-, depression-like behavior and social deficits in adulthood.
Garcia-Valtanen (2020) [62]	C57Bl/6J	GD12.5	20 mg/kg poly(I: C)	GD18	Placenta	Il-6, Il-10, Il-12(p40), Tnfα	
					Brain	Il-12(p40)	
				PND7	In vitro splenocytes	Il-1β, Il-10, Tnfα	± Stimulation.
					In vitro liver	Il-10, Il-6, Tnfα	± Stimulation.
					In vitro brain	Il-1β	± Stimulation. Anxiety-like behavior and social deficits after LPS immune induction.
Shimizu (2021) [72]	C57Bl/6J	GD12.5, 14.5, 16.5	20 mg/kg poly (I: C)	PND24	Serum	Il-6, Il-17, Infγ	24 h after postnatal immune induction
Rose (2017) [71]	Rhesus monkey	GD43-46 GD100-103	0,25 mg/kg poly(I: C)	PND400, PND1360	Plasma	Il-2, Il-6, Il-10, Il-13, Ifnγ, Tnfα, G-CSF, G-CSF, Cxcl8, Mcp-1	
					In vitro PBMCs	Il-1β, Il-2, Il-4, Il-6, Il-10, Il-12(p40), Il-17, Tnfα, Ifnγ, G-CSF, GM-CSF, Mip-1α, Mip-1β, Mcp-1, Cxcl8	± TLR-3 or TLR-4 stimulation.
Surriga (2009) [90]	Sprague Dawley	GD18	500ug/kg LPS	PND21	Liver	Il-6	In vivo LPS stimulation.
Talukdar (2021) [85]	Sprague Dawley	GD12.5	20 mg/kg poly(I: C) or 1,5 mg/kg LPS	PND60	Brain	Il-1β, Il-18	Hippocampus. Anxiety-like behavior and social deficits in adolescents and adulthood.
Brown (2022) [70]	Wistar rats	GD19	4 mg/kg poly(I: C)	PND85	Serum	No alterations in cytokine profile	

**Table 2:** Immune cell profile and immune function alterations observed in MIA exposed offspring. Reported alterations in the immune cell profile and immune function in different cell populations or after postnatal secondary hit from multiple studies. GD; gestational day, PND; postnatal day, EAE; experimental autoimmune encephalomyelitis

References	Model	MIA induction		Age offspring	Immune profile/function	Cell type/ secondary hit	Observed outcomes
		GD	Method				
Hsiao (2012) [77]	C57Bl/6 N	GD12.5	20 mg/kg poly(I: C)	Adult*	Immune cell profile	Splenocytes	Decrease in CD4^+^Foxp3^+^ in splenocytes
							Decrease in CD4^+^Foxp3^+^CD25^+^ Regulatory T cells
						HSCs & progenitors	Elevation peripheral Gr-1^+^ cells, not for other major lineages
							Differentiation into granulocyte precursors (CFU-G) increased and decreased for granulocyte-macrophage precursors (CFU-GM)
				GD13.5, GD15.5			Altered myeloid lineage potential and differentiation of fetal liver HSCs/progenitors
Luan (2015) [73]	C57Bl/6J	GD12.5	50ug/kg LPS	PND14	Immune cell profile	Thymocytes	No differences in CD4^+^ and CD8^+^ T cells
				PND70		Splenocytes	Increase in CD4^+^Foxp3^+^ T cells
				PND56-PND70	Immune function	CD4 + T cells	Increased proliferation and survival rate naïve T cells
							Upregulation genes involved in immune related processes
						Splenocytes	Elevation in Il-17a^+^Ifny^+^, Il-17a^+^Tnfa^−^, Il-17a^+^Infy^−^ and Il-17a^+^RORyt^+^ producing CD4^+^T cells
						Hepatocytes	Elevation in Il-17a^+^Tnfa^−^and Il-17a^+^Infy^−^ producing CD4^+^ T cells
						Sensitivity to LPS	Hyperreactivity with decreased survival rate
Mandal (2010/2011) [86, 87]	C57Bl/6J	GD12.5	20 mg/kg poly(I: C)	PND20 -PND30	Immune cell profile	Splenocytes	Preferential differentiation toward Th17 cells
Mandal (2013) [74]	C57Bl/6J	GD12.5	10 mg/kg poly(I: C)	PND60	Immune cell profile	Splenocytes	Preferential differentiation toward Th17 cells
					Immune function	Acute inflammation	Increase peritoneal exudate cells
							Increase Il6, Tnfα and Il-10 in peritoneal fluids
						EAE model	Earlier onset EAE, with partial or total tial paresis 1 day after EAE induction
							High disease incidence until day 4 for MIA offspring
Giulivi (2013) [89]	C57Bl/6J	GD12.5	20 mg/kg poly(I: C)	PND84	Immune function	Splenocytes	Lower complex I activity for mitochondrial ATP production
Hsueh (2018) [65]	C57Bl/6J	GD15 -GD17	25-50ug/kg LPS	PND56	Immune function	Sensitivity to LPS	After LPS stimulation an increase in cytokine, chemokines and CAMs in plasma
Lim (2021) [88]	SPF C57Bl/6j	GD10.5	200ul suspention *Yersinia psuedotuberculosis* (*yopM* - oral)	PND35-56	Immune cell profile	Small & large intestinal lamina propria	Increase Th17 cells
López (2022) [91]	C57Bl/6J	GD14.5	20 mg/kg poly(I: C)	GD15.5	Immune function	HSCs & progenitors	Increase in CD45^+^ HSCPs
							Upregulation inflammatory gene profile in HSC population
							Increase cellularity & hyperresponsiveness in fetal-derived innate-like lymphocytes
				GD15.5-GD17.5			Expansion & persistence of fetal lymphoid-biased progenitors
López (2023) [92]	C57Bl/6J	GD10.5	*Toxoplasma gondii*	GD16.5	Immune function	HSCs & progenitors	Virulence-dependent effect on proliferation, self-renewal and lineage output HSC
Loayza (2023) [93]	C57Bl/6J	GD12.5	50ug/kg LPS	GD15.5 - PND21	Immune function	Microglia	Increased number microglia in neurogenic regions. Alterations microglial phenotype and morphology.

* Specific age was not mentioned

### Effect of MIA on the cytokine profile and production

Multiple studies have analyzed the cytokine profile of offspring exposed to *in utero* MIA using various models, at different ages of the offspring, and in different sample types. Table 1 provides an overview of the reported alterations in the cytokine profile. The and online dataset is a searchable spreadsheet, which provides a more thorough summary of the cytokine profile, including the analyzed cytokines, the MIA model utilized, the age of the offspring, and the specific sample used. An acute effect of MIA was observed in the placenta after 48 h, with elevated levels of tumor necrosis factor alpha (Tnfα), interleukin (Il)-6, Il-12(p40) and a reduction in Il-10 [62].

### Effect of MIA on the cytokine profile in the offsprings’ serum and plasma

Alterations in the cytokine profile in serum and plasma have been reported for different ages and models of the MIA exposed offspring. In the plasma of offspring exposed to poly (I: C), an elevation in Il-2, Il-5, and Il-6 was observed at postnatal day (PND)10, while no differences were observed for offspring exposed to LPS induced MIA [63]. Garay and colleagues [64] analyzed the serum cytokine profile of MIA exposed offspring from birth until adulthood. The cytokine profiles at birth, PND7, PND14, and PND30 were altered. However, alterations did not persist until adulthood. Moreover, the alterations found in the cytokine profile were not consistent during development [64]. In the serum of MIA exposed offspring, increases in Il-12(p40) and RANTES (regulated upon activation normal T cell expressed and secreted) and decreased levels of Il-3, granulocyte-macrophage colony-stimulated factor (GM-CSF), and macrophage inflammatory protein (Mip)-1α were reported at birth. A different profile was present at PND7, with an increase in Il-1β, Il-3, Il-6, Il-12(p40), granulocyte colony-stimulating factor (G-CSF), interferon (Ifn)γ, RANTES, and Tnfα, and a decrease was found for Il-1α, Il-2, and Il-12(p70). All the cytokine levels, except for in Tnfα and Mip-1β, were back to baseline levels at PND14. There was an elevation in Il-1β, Il-6, and Il-9 and a decrease in Il-3 reported on PND30, while no differences in cytokine levels were found in adulthood (PND60) [64]. Others analyzed the serum or plasma cytokine profile in both adolescent and adult offspring. An elevation in Il-1β [65, 66] and Il-6 [65] was observed in adolescents, and an elevation in Il-1β [65–67], and Il-10 [65] was observed in adult offspring exposed to MIA. In contrast, a decrease in Il-6 and Tnfα was also reported in adolescent offspring, with a decrease in Il-2 and Ifnγ in adult offspring exposed to MIA [68]. Furthermore, no alterations in plasma cytokine profiles have been reported in adolescent and adult offspring [67, 69].

In a Wistar rat model, no differences in the serum cytokine profile were reported in MIA exposed adult offspring [70]. Alterations in cytokine production were demonstrated in a non-human primate model [71]. Serum concentrations of innate inflammatory cytokines Il-1β, Il-6, Il-12(p40), and Tnfα were elevated in one year old MIA exposed offspring. Il-1β levels remained elevated in the serum of 4-year-old MIA exposed offspring. In addition, concentrations of Th2 cytokines Il-4 and Il-13 were increased in MIA offspring at the age of 4 years [71].

After postnatal immune induction, alterations in the serum cytokine profile were reported in offspring exposed to MIA. *Ifng* gene expression was upregulated in 3–4 weeks old offspring exposed to *in utero* MIA. Moreover, serum Il-6 and Il-17 cytokines were increased 24 h after postnatal poly (I: C) injection [72]. Following LPS-induced shock, a dose-dependent increase in serum Tnfα was reported in adult MIA exposed offspring [73]. Hsueh and colleagues [65] observed an increase in Il-1β, Il-6, and Il-10 in the serum of adult MIA offspring. After immune induction with LPS, various cytokines, chemokines, and cell adhesion molecules (CAM) were elevated in the serum of adult MIA exposed offspring when compared to control offspring. For the cytokines, an increase in Tnfα, Il-1β, Il-6, Il-10, Il-12p40, Il-17a, and Ifnγ was observed. An increase in the chemokines monocyte chemoattractant protein (Mcp)-1, monokine induced by interferon gamma (Mig), Mip-1α, Mip-1β, and RANTES was reported. Elevation in the cell adhesion molecules L-selectin, P-selectin, and intracellular adhesion molecule (Icam)-1 was observed [65]. After immune induction with zymosan, a significantly higher amount of Il-6, Tnfα, and Il-10 was present in the peritoneal fluid of the MIA exposed offspring [74, 75].

In summary, the observed alterations in the serum and plasma cytokine profiles vary considerably in MIA exposed offspring. The cytokine profile varies not only between the ages of the offspring but also between the studies. Moreover, multiple studies reported more non-affected cytokine concentrations than altered cytokine concentrations. The above-described studies have different timing of induction of MIA, with mid-gestation and late-gestation induction of MIA. A systematic review with meta-analysis compared the timing, age of offspring and the effect of MIA on cytokine concentrations. An increase in Il-6 was observed for MIA exposed offspring, with a bigger impact for mid gestation poly (I: C) MIA induction. Furthermore, differences in Il-1β, Il-10 and Tnfα were reported without associations with the offspring’s age and gestational induction period [76].

### Effect of MIA on in vitro cytokine production

Differences in cytokine production have also been observed in vitro for several cell types obtained from the offspring of murine MIA models. In vitro cultures of neonatal splenocytes (PND7) showed an increase in Il-1β and Tnfα and a decrease in Il-10 production [62]. The same increase in Il-1β and Tnfα production and decreased Il-10 production were observed when the splenocytes were stimulated with LPS [62]. An increased production of cytokines was observed for CD4^+^ T cells originating from the spleen and mesenteric lymph nodes of adult offspring. Furthermore, in vitro stimulation of the CD4^+^ T cells resulted in elevated production of Il-6 and Il-17 [77]. CD4^+^ T cells of MIA exposed offspring showed elevated Th1/Th17 cytokine production from the spleen and Th17 cytokine production from the liver [73]. Bone marrow derived macrophages of MIA exposed adult offspring produced more Il-12(p40) and Mip-1α when LPS was added to the in vitro culture [78]. This increased Il-12(p40) and Mip-1α was also observed for polarized M1 macrophages stimulated with LPS [74]. Polarization toward M2 macrophages with LPS stimulation resulted in an increase in Il-1β and Mip-1α cytokine production [78].

Peripheral blood mononuclear cells (PBMC) in vitro culture from the non-human primate model showed elevated cytokine production for 1- and 4-year old MIA exposed offspring [71]. Elevated production of Il-6, Il-12(p40), Tnfα, G-CSF, GM-CSF, Mip-1α, and Mip-1β was found at baseline and after stimulation in PBMC culture of 1-year old MIA exposed offspring. At 4-years of age, an elevated production of Il-1β, G-CSF, and Mcp-1 was observed at baseline and after immune stimulation. There are similarities between plasma and in vitro PBMC cytokine production for the MIA exposed offspring at 1 year old. Both profiles demonstrate an increase in Il-2, Tnfα, Ifnγ, and G-CSF. However, the production of 11 cytokines was increased in the in vitro PBMC culture, while only an increase for 5 cytokines was found in the plasma [71]. The same applies to the plasma and in vitro PBMC cytokine production assessed at the age of 4 years, with an increase for Il-10, Cxcl8, and Mcp-1 in both profiles. An increased production of 6 cytokines was observed in the in vitro PBMC culture, while five cytokines were increased in the plasma [71].

### Effect of MIA on the cytokine profile in the offspring’s brain

As previously stated, most studies using preclinical MIA models concentrate on the association between MIA and neurodevelopmental disorders. Numerous studies have analyzed the cytokine profile in the brains of the offspring at various ages and brain regions. (Tables 1 and online dataset). The cytokine patterns in the brain of the offspring were affected by the timing, severity, and interval between induction and analysis of MIA throughout gestation [79]. A meta-analysis of the offspring’s cytokine levels, however, did not support this [76].

The cytokine profile in the offspring’s brain was examined as early as three hours following MIA induction. In the fetal brain, three hours after mid-gestation (GD9) MIA induction, an increase in Il-6 and a decrease in Il-1β and Il-10 was observed. Six hours after MIA induction, an elevation in Il-1β and Il-6 was observed [79]. The fetal brain of offspring exposed to late gestational MIA (GD17) showed the opposite cytokine profile, with a decrease in Il-6 and an increase in ll-1β and Il-10 three hours after MIA exposure. The only change seen six hours after late gestational MIA was an elevation in Il-6 [79]. Alterations were observed in the fetal brain for pro-inflammatory cytokines, chemokines, and colony stimulating factors six and 24 h after MIA induction on GD16 [80]. 48 h after MIA induction on GD12, an increase in Il-12(p40) [62], Il-17α and Il-6 [81] was observed in the fetal brain. Interestingly, oral probiotic administration during pregnancy prevented the increase of Il-17α and Il-6 in the fetal brain [81]. Induction of MIA between GD15 and GD17 resulted in an elevation of Tnfα in the fetal brain on GD18, while no differences were observed at PND10 [63].

Besides the fetal and neonatal brains, the cytokine profiles of MIA exposed offspring have also been analyzed in the adolescent and adult brains. Multiple studies analyzed the cytokine profile in the offspring brain for different brain regions during development, revealing alterations in the brain cytokine profile for offspring exposed to MIA throughout development that were region-specific [64, 82]. Garay and colleagues [64] analyzed the cytokine profile in the frontal cortex, cingulate cortex, and hippocampus from birth until adulthood, besides the previously mentioned serum cytokine profile. In the frontal cortex, 18 different cytokines were altered during development. In the cingulate cortex, 17 different cytokines were altered throughout development. None of the cytokines were altered at all ages. In the hippocampus, 14 different cytokines were altered at birth or during development [60]. Overall, cytokine levels were elevated at birth, reduced during postnatal brain development, and increased in adulthood in the frontal and cingulate cortex of MIA exposed offspring. The cytokine alterations in the hippocampus had a mixed profile, with both increases and decreases between the ages. There were no similarities between the serum and region-specific cytokine profiles in MIA exposed offspring. The cytokine profiles from the serum and the region-specific brain varied [64]. The cytokine profile in the hippocampus of MIA exposed offspring was analyzed in multiple studies. In adult offspring, an increase in Il-1β was reported [67]. Nevertheless, no persistent systemic inflammation was observed in the hippocampus of adolescent and adult offspring exposed to *in utero* MIA [67]. In aged MIA exposed offspring, an increase in Il-1β and Il-6 was reported in the hippocampus [66]. Following postnatal immune induction, an increase in several cytokines, chemokines, and cell adhesion molecules was reported in the brains of adult MIA exposed offspring [65]. In contrast, no alterations in pro- and anti-inflammatory cytokines were reported in the hippocampus of adolescent, adult, and aged offspring exposed to GD17 induced MIA [69]. The cytokine profile in the cerebellum of MIA exposed offspring from birth until adolescence was also analyzed [82]. The cytokine profile in the cerebellum of MIA exposed offspring was mixed, with increases, decreases, and no differences in levels of different cytokines at different ages [82].

Furthermore, sex-specific and brain-region specific alterations were reported. In female adult MIA exposed offspring, an increase in Il-1β, Il-6 was observed in the amygdala and an increase in Tgf-β was observed in the hippocampus, while no alterations were found in the male MIA exposed offspring brain [83]. In both male and female offspring exposed to MIA, Il-10 was altered in the medial prefrontal cortex and hippocampus. However, in male offspring a decrease in Il-10 was observed, while an increase was found for female offspring [83].

In summary, alterations in the cytokine profile have been reported in the MIA exposed offspring’s brain. The cytokine profiles do not only vary between studies but are also region-specific and sex-specific. A thorough summary of the cytokine profile can be found in the and online dataset.

### Effect of MIA on the immune related transcriptional profile in the offspring’s brain

Along with the differences in cytokine profiles, there were also differences reported for immune-related genes in the brains of MIA exposed offspring. Following MIA induction on GD12, differences in gene expression of *Il1b*,* Il6*,* Tnfa*,* Il10*, and *Mcp1* were observed during brain development [84]. The differences in gene expression varied between the ages of the offspring [84]. Another study reported that MIA induced alterations in the gene expression of genes associated with inflammatory signaling, Il-2 stimulated pathways, selectin, developmental signaling, hormones, and synaptic structure were upregulated [65]. In a rat model, altered gene expression was observed in the hippocampus of adult MIA exposed offspring. For both LPS and poly I: C induced MIA, an increase in gene expression was observed for *Il1b* and *Il18* in the hippocampus. Furthermore, an increase in gene expression was found for toll-like receptors (TLRs) and inflammasome pathway related genes [85].

Carlezon and colleagues [83] identified alterations in gene expression in the brains of MIA exposed offspring, in addition to previously mentioned alterations in protein cytokine profiles that were sex and brain region-specific. In female MIA exposed adult offspring, there was an increase in *ll1b* and *Il6* gene expression observed in the medial frontal cortex, amygdala, and hippocampus. However, only an increase in Il-1β and Il-6 proteins was observed in the amygdala. An increase in Tgf-β1 was observed in the hippocampus of female offspring, which corresponds with the observed elevated gene expression [83]. In both male and female offspring, differences in anti-inflammatory factor *Il10* gene expression were observed in the medial prefrontal cortex and hippocampus. *Il10* gene expression was higher in females and lower in male MIA exposed offspring. This was consistent with the findings of altered Il-10 protein in both male and female offspring [83]. While there was no change in the IL-6 protein expression in male offspring, increased *Il6* gene expression was observed in the medial prefrontal cortex and amygdala [83]. Male MIA exposed offspring had elevated *Tnfa* gene expression in the medial prefrontal cortex and thalamus. The *Tnfa* gene expression was elevated in the hippocampus of the female MIA exposed offspring [83].

In summary, alterations in the gene expression of cytokines have been reported in the MIA exposed offspring’s brain.

## Effect of MIA on offsprings immune cell profile

Alterations in the peripheral immune cell profile have been observed for offspring exposed to MIA and are summarized in Table 2. A few studies demonstrated differences in cell composition in the MIA offspring. Contradicting results were reported for CD4^+^Foxp3^+^ splenocytes in adult offspring exposed to MIA, with both elevated and reduced levels being reported [73, 77]. Also, an increase in CD4^+^Foxp3^+^CD25^+^ regulatory T (Treg) cells was reported in splenocytes [69]. The splenocytes from offspring exposed to MIA from dams immunized before pregnancy showed preferential differentiation into Th17 cells [74, 86, 87]. Furthermore, an elevation in peripheral Gr-1^+^ cells was described, suggesting a skewing in the hematopoietic stem cells (HSC) or progenitor cells toward neutrophils and monocytes [77]. A colony-forming assay demonstrated increased differentiation into granulocyte precursors (CFU-G), and decreased differentiation into early granulocyte-macrophage precursors (CFU-GM). Similar results were observed for the colony-forming assay of fetal liver HSCs and progenitors of MIA offspring, showing an altered myeloid lineage potential and differentiation of the MIA offspring [77]. Alterations in the immune profile were found in the small and large intestinal lamina propria, with an increase in Th17 cells showing that MIA can affect tissue-specific immunity [88].

In summary, alterations in the immune cell profile have been observed in four studies. However, contradictory results have been reported on these alterations in the immune cell profile.

## Effect of MIA on immune cell function

Besides differences in cytokine production and immune cell profile, alterations in immune function have been described (Table 2). Along with the previously described alterations in cytokine production, an increase in proliferation and an enhanced survival rate of naïve CD4^+^ T cells from MIA offspring were observed [73]. Transcriptomic analysis of resting CD4^+^ T cells from MIA offspring showed an upregulation in genes involved in immune-related processes when compared to offspring from control dams. Proteomic analysis showed 18 upregulated proteins in resting CD4^+^ T cells of MIA offspring, most of which belonged to metabolic processes. For stimulated CD4^+^ T cells, five proteins belonging to metabolic process were upregulated in MIA offspring [73]. Long-lasting effects on the bioenergetics of splenocytes were observed in adult offspring exposed to MIA. A lower complex I activity for mitochondrial ATP was observed, resulting in a decrease in mitochondrial ATP production and oxygen uptake. The splenocytes of MIA offspring seem to prefer fatty acids over glucose as the main substrate for mitochondrial oxidative phosphorylation [89]. Alterations in the hepatic inflammatory response were observed, with a lower *Il6* gene expression and a decrease in p42/44 MAPK phosphorylation after LPS stimulation at PND21 for MIA exposed offspring. The gene expression of *Il6* correlated with the decreased phosphorylation of p42/44 MAPK, indicating an important role for p42/44 MAPK in the regulation of hepatic Il-6 expression [90].

Adult HSCs are quiescent and produce the appropriate number of lineage-biased-multipotent progenitors. In response to inflammation, HSPCs exit the quiescent state, mobilize, and differentiate into mature myeloid cells in response to infection. However, little is known about the response of fetal HSCs to inflammation [51]. A response of fetal HSCs and progenitor cells to MIA, which shaped postnatal hematopoiesis and immune cell function was reported [91]. An increase in proliferation and persistence of fetal lymphoid-biased progenitors was observed in MIA exposed offspring. Single-cell transcriptomic analysis demonstrated an increase in inflammatory gene profiles, promoting lymphoid-biased progenitors in discrete and transient HSC populations. At the same time, an increase in cellularity and hyperresponsiveness was seen in fetal-derived innate-like lymphocytes [91]. In a follow-up study, the effect of *Toxoplasma gondii* induced MIA on fetal hematopoiesis was analyzed [92]. *T.gondii* induces Type II IFNγ-mediated immune activation, while the previously described poly (I: C) MIA is induced by Type I IFN signaling. Fetal hematopoiesis was affected in a virulence-dependent manner, with alterations in proliferation, self-renewal potential, and lineage output. The severity of the MIA appears to drive alterations in fetal hematopoiesis by triggering HSC proliferation and the expansion of downstream HSCPs in the fetal liver [92].

In summary, beside alterations in cytokine production, differences in transcriptomic, proteomic, cell survival, and function of the immune cells have been reported. Furthermore, early effects of MIA were reported, with alterations in fetal hematopoiesis. However, there are only a few studies that have investigated these alterations.

### Effect of MIA on the microglia

Microglia cells originate the EMPs generated in the yolk sac. The primitive macrophages migrate the brain between GD8.5-9.5 (4.5 weeks in humans). This is before the establishment of the BBB around GD13. These primitive macrophages give rise to the microglia [54]. Alterations in the cytokine profile also affect the microglia. More activation of microglia was observed after an immune challenge in the brain of MIA exposed offspring [66]. An increase in the protein level of CD68 was found in the fetal brain of MIA exposed offspring, suggesting an increased activation of microglia [63]. Furthermore, an increase in metabotropic glutamate receptor subunit 5 (mGluR5) was observed in the fetal and PND10 brains of MIA exposed offspring, which is associated with neuroinflammation [63]. In neurogenic regions, MIA exposure increased the number of microglia in fetal and neonatal offspring [93]. The brains of both fetal and neonatal offspring exposed to MIA showed alterations in the microglial phenotypes. In the subventricular zone, these alterations led to excessive proliferation and an overabundance of neural progenitors [93]. Furthermore, changes in the microglial morphology have been observed in the developing brain, GD12 till birth, and postnatally in the amygdala, PND7 till PND40, of MIA exposed offspring, suggesting long-term microglial activation [84]. The effect of MIA on the microglia and the neurodevelopment has been recently reviewed by others [94].

## Effect of MIA on immune induction

As described earlier, differences in cytokine profiles were found after postnatal immune induction for MIA exposed offspring. The observed alterations in cytokine profiles after postnatal immune induction in the serum and plasma varied considerably [65, 72–75]. An increased sensitivity to LPS treatment for MIA exposed offspring was reported, along with an increase in serum Tnfα in adult offspring after LPS-induced shock [73]. Besides this increase in Tnfα, an hyperreactivity was observed for the LPS-induced shock, with a decrease in survival rate for MIA exposed offspring [73].

Mandal and colleagues [74] analyzed the immune response of adult offspring exposed to *in utero* MIA with a second hit: an acute inflammatory response model and an experimental autoimmune encephalomyelitis (EAE) model. In the acute inflammation model, the non-specific inflammatory response was induced, and the nature of the inflammatory response was assessed. In adult offspring, a 2-fold increase of peritoneal exudate cells, mostly consisting of neutrophils, was observed. Furthermore, a significantly higher amount of Il-6, Tnfα and Il-10 was present in the peritoneal fluid of the MIA exposed offspring [74, 75]. In the EAE model, mice received the synthesized encephalitogenic peptide myelin oligodendrocyte glycoprotein, stimulating an antigen-specific T cell response. This model is used to examine the adaptive immune system, and the model is commonly used for the human inflammatory demyelination disease multiple sclerosis (MS) [74, 95]. In the EAE model, an earlier onset of EAE was observed in MIA offspring, with signs of clinical deficits as early as 24 h. Partial or total tail paresis on day 1 manifested for 45% of the offspring, and disease incidence was higher in the MIA offspring until day four [74, 75]. In MIA exposed adult offspring, the innate and adaptive immune responses may react more strongly when exposed to stimuli, as demonstrated by the acute inflammatory response and EAE models.

## Offsprings behavior after MIA exposure associated with immune outcomes

The majority of studies focusing on the effect of MIA on neurodevelopment and immune outcomes in the brain include behavioral analyses of the offspring. Differences in behavioral outcomes were seen with mid- and late-gestational induction of MIA. Offspring exposed to mid-gestational MIA showed reduced open-field exploration, while late-gestational MIA led to perseverative behavior in adult mice [79]. Interestingly, cognitive and behavioral abnormalities seem to be corelated with the duration of poly(I: C) exposure. Around GD9, exposure to poly(I: C) is associated with impairments in sensorimotor gating and decreased expression of dopamine D1 receptor in the prefrontal cortex. Exposure at GD17 is associated with reduced expression of NMDA-receptor subunit-1 in the hippocampus and impaired working memory [96–98]. The establishment of the BBB may relate to these variations. The primitive macrophages, the precursors of the microglia, migrate to the brain prior to establishment of the BBB [54]. Thus, early gestational poly(I: C) administration may impact the migrating microglia cells.

A non-human primate MIA model showed altered behavioral phenotypes. The production of innate cytokines in the first two years of life was associated with stereotyped behavior. Production of Th2 cytokines at the age of four was linked to self-directed behavior in the MIA exposed offspring [71]. Overall, in adolescence and adulthood, more anxiety-like behaviors [62, 65, 81, 85], depression-like behavior [81], social behavioral deficits [62, 65, 81, 83, 85], and repetitive behaviors [81, 83] were observed in MIA offspring, associated with *in utero* MIA exposure. Wang et al. reported that anxiety-like and depression-like behavior, social deficits, and repetitive and stereotyped behaviors observed in adult MIA exposed offspring can be prevented with oral probiotic administration during pregnancy. This was reported in combination with decreased levels of Il-6 and Il-17α protein in the fetal brains, which were elevated without probiotic administration [81].

## Fetal programming effect of MIA on the immune system

**Fig. 2:**
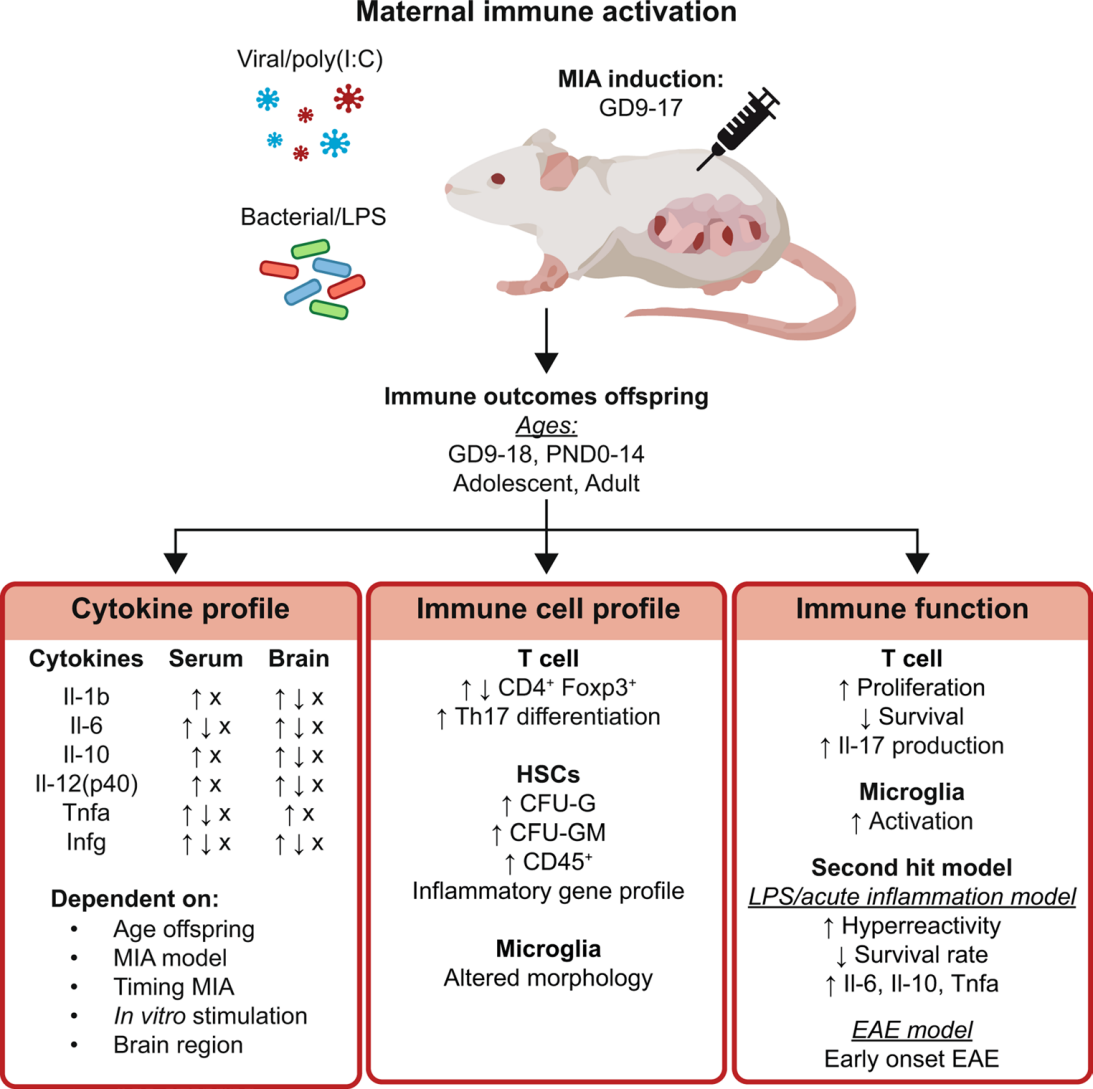
Overview of reported alterations in the offspring’s immune system. The bacterial mimic LPS and the viral mimic poly(I: C) are commonly used to induce MIA in preclinical models, with induction between GD9 and GD17. The immune outcomes reported were for the offspring are measured at difference ages. The alterations found in the immune outcomes varies between studies, timing, MIA model, brain region and offsprings age. Details on the cytokine profile can be found in Table 1 and **online dataset.** Details on the immune cell profile and function can be found in Table 2. ↑; increased, ↓; decreased, X; no differences, CFU-G; colony-forming unit granulocyte progenitors, CFU-GM; colony-forming unit granulocyte-macrophage progenitors, EAE; experimental autoimmune encephalomyelitis

Overall, reports of alterations in immune cell function and profile, as well as cytokine production and profile, suggest that MIA may affect the fetal immune system (Fig. 2). There is evidence of an inflammatory phenotype, which can trigger an increased inflammatory response in adulthood. However, it is crucial to keep in mind that the reported alterations are varying, contradicting and negative results have also frequently been reported (and online dataset).

Considering the critical windows of immune development (Fig. 1), the difference in timing of MIA induction might explain the variation in the reported results. The described studies induced MIA at mid- or late-gestation, both falling within the second window of critical immune development of hepatic hematopoiesis. Mid-gestation MIA induction is at the start, while late-gestation MIA induction is at the end of this second window of critical immune development. During hepatic hematopoiesis, the HSCs and progenitors are located in the fetal liver, which is the primary site of hematopoiesis after GD10 in mice. During this period, the definitive HSCs undergo proliferation and differentiation [46, 47, 50, 51], which could be affected by mid-gestational induction of MIA. The HSCs migrate around GD16.5 to the bone marrow [47, 55, 57, 99]. Late-gestational induction of MIA might affect the migrated HSCs in the bone marrow and the progenitors located in the other immune organs. This might explain the variation reported on the fetal programming effect in MIA models. However, as mentioned earlier, the timing of the MIA was only associated with Il-6, while reported differences in Il-1β, Il-10, and Tnfα were not associated with the timing of the MIA induction [76].

## MIA in humans: observations from the clinic

Since the COVID-19 pandemic, more research has focused on the possible health effects of *in utero* exposure to MIA in humans. Recently, the effect of COVID-19 exposure *in utero* on the child’s health was reported. A few studies analyzed the cytokine and immune cell profiles of cord blood at delivery [100–102]. A significant increase in Il-10 was observed in cord blood plasma from neonates born to mothers with a recent or ongoing SARS-CoV-2 infection [100]. Others reported a mild cytokine response, with only an increase in Il-8 in the cord blood [101]. In the peripheral serum, increases in Il-6, Il-8, and interferon gamma-induced protein (IP-10) were found in infants born to mothers with SARS-CoV-2 infection during pregnancy [103]. Increases in Il-1β, Il-6, and Il-8 were observed in an in vitro cord blood mononuclear cell culture with and without immune stimulation [102]. In contrast to these studies, no differences in peripheral serum cytokine levels were also reported in neonates born to mothers with SARS-CoV-2 infection during pregnancy [104].

When looking at the immune cell profile in cord blood, no major differences were observed [104]. In addition, no differences were observed in the lymphocyte subsets for neonates born to mothers with SARS-CoV-2 infection during the third trimester. Furthermore, no differences in cellular and humoral immunity were reported [104]. Others reported no differences in the B cell, CD4^+^, and CD8^+^ T cell populations [100]. However, specific alterations in the adaptive cell population were observed. An increase in regulatory T cells was found in neonates born to mothers with a recent or ongoing SARS-CoV-2 infection. In addition, an increased percentage of innate immune cells, natural killer (NK) cells, in neonates born to mothers with recent or ongoing infection was present. Furthermore, an increased percentage of cytokine-producing cells was observed in neonates born to mothers exposed to SARS-CoV-2 [100]. Immune cell subset specific differences in cytokine expression have been described in cord blood mononuclear cells [102]. Garcia-Flores and colleagues [101] reported no differences in the immunophenotype of cord blood T cells. However, differences in transcription have been observed in the cord blood, with 131 upregulated and 294 downregulated genes. In the upregulated genes, an enrichment in biological processes for defense responses to fungus and bacteria was observed [101]. Matute and colleagues [105] characterized the fetal immunologic landscape in pregnancies complicated by mild maternal SARS-CoV-2 infection. Alterations in the transcriptional pattern were observed in the cord blood mononuclear cells of newborns from SARS-CoV-2 infected mothers. An increase in gene expression of IFN-stimulated and major histocompatibility complex genes was observed in CD14^+^ monocytes. Gene Ontology enrichment demonstrated enrichment for genes associated with antigen processing and presentation, regulation of neutrophil differentiation, and viral translational termination and reinitiation [105]. In the cord blood of SARS-CoV-2 positive pregnancies, the transcriptional alterations of NK cells suggest activation and exhaustion. An upregulation in IFN-stimulated genes was observed, with an increase in the expression of *CCL4* (MIP-1β) and cytotoxic genes in NK cells. Differentially expressed genes were enriched for genes related to antigen processing and presentation, regulation of adaptive immune memory response, INF-α response, regulation of NK cell cytokine production, and viral transcription [105]. The transcriptional alterations in B cells suggest a potential B cell dysfunction, with a decrease in the transcription of genes downstream of the B cell receptor. In addition, fetal T cell clonal expansion was observed [105]. Furthermore, an increase in IgG was observed in the cord blood of neonates from mothers with SARS-CoV-2 infection [100]. In contrast, no differences were identified in IgG and IgM levels in the peripheral serum of neonates [104].

## MIA, neurodevelopmental disorders, and the fetal immune system

As discussed in the introduction, an increasing body of epidemiological evidence suggests an association between maternal infection, maternal immune activation, and the increased risk of neurodevelopmental disorders [12–24]. Furthermore, abnormal immune responses have been observed in neurodevelopmental and neuropsychiatric disorders [28–33]. There is a high incidence of an aberrant immune response in individuals with autism and schizophrenia [23, 28], including increased numbers of monocytes [29, 34] and alterations in the plasma cytokine profile [31, 32, 35]. These studies link immune dysfunction to neurodevelopmental disorders.

## Future perspective & conclusion

Fetal programming of the immune system has received increased attention in the last couple of years. Keeping in mind that adult HSCs are generated in the first trimester in humans, exposure to adverse circumstances like pro-inflammatory cytokines from MIA might influence their function in later life. Furthermore, the HSCs and the multipotent progenitor cells undergo rapid proliferation before migration to the bone marrow, creating the second critical window of development. The last three critical windows, from the second trimester until around 5 years after birth in humans, consist of the expansion of the lymphocytic cell population, functional development, and maturation of the immune system. Adverse circumstances during these windows can also influence the immune response in later life, maybe not by affecting the HSCs, but by affecting the progenitor cells.

Most of the pre-clinical MIA models focus on the association with neurodevelopmental disorders, while some studies report effects on the immune system. However, the observed alterations in the immune response and system differ from each other and are contradictory. Furthermore, negative results have also been frequently reported. The differences between the results might be explained by the model used, the timing of the MIA induction, the timing of the analysis, and the selected tissue for analysis. There is a clear need for more research on the non-neurodevelopmental consequences of MIA, including but not limited to later immune function. A secondary immunological challenge in later life might be necessary to evaluate the consequences of MIA on the offsprings immune system.

Due to the COVID-19 pandemic, there has been an increase in research focusing on the possible long-term health effects of *in utero* exposure to MIA by viruses. A few studies analyzed the immune state of the neonates born to mothers with a SARS-CoV-2 infection. Furthermore, results suggest that COVID-19 exposure *in utero* may be associated with neurodevelopmental changes [106]. Male infants born to mothers who tested positive for SARS-CoV-2 during pregnancy had a higher chance of receiving a neurodevelopmental diagnosis within the first year [106].

Overall, there is a limited amount of research focusing on the effect of MIA on the immune function of the offspring. Due to the limited research available, no ultimate conclusions can be drawn regarding the extent, direction, and duration of the fetal programming effect of MIA. However, there are indications that MIA may be able to program the fetal immune system, as seen in alterations in the cytokine profile, immune cell profile, and function. Epigenetic modifications are generally considered the mechanism for fetal programming. The epigenetic programming of the immune system is important for the maintenance, tolerance, training, and memory of the immune response. Therefore, alterations in the epigenetic markers, including histone modifications and DNA methylation, in the immune cells can influence the immune system [10, 11].

Future research focusing on the offspring’s immune function after MIA exposure can elucidate pathways that may contribute to fetal immune programming and long-term health outcomes. A secondary immunological challenge in later life might be necessary to evaluate whether the immune system of the offspring is affected by early life MIA exposure.

## Data Availability

The generated dataset is available in the Zenodo repository, 10.5281/zenodo.13354923.
